# Celecoxib: Not So Harmless?

**DOI:** 10.7759/cureus.113247

**Published:** 2026-07-23

**Authors:** Ousmane Kaba, Bouchra Chahboun, Maman Idi Chazali, Houssam Bkiyar, Brahim Housni

**Affiliations:** 1 Department of Anesthesiology and Intensive Care Unit, Mohammed VI University Hospital, Oujda, MAR; 2 Department of Anesthesiology and Intensive Care Unit, Faculty of Medicine and Pharmacy of Oujda, Mohammed First University, Oujda, MAR

**Keywords:** adverse drug reactions, celecoxib, therapeutic emergency, toxic epidermal necrolysis, toxidermia

## Abstract

Toxic epidermal necrolysis (TEN) is a severe and potentially fatal cutaneous reaction secondary to the use of drugs, including celecoxib. Celecoxib is a nonsteroidal anti-inflammatory drug (NSAID) that is widely prescribed to relieve arthralgia because of its better gastrointestinal tolerance. However, it may also be responsible for skin reactions, most of which are benign. Serious cutaneous adverse reactions have been reported in the form of acute generalized exanthematous pustulosis (AGEP). TEN secondary to celecoxib is poorly documented.

Discontinuation of the suspected or causative drug(s), transfer of the patient to a specialist unit as quickly as possible, and supportive care are the cornerstones of patient management.

## Introduction

A severe and potentially life-threatening cutaneous reaction known as toxic epidermal necrolysis (TEN) may occur as a result of drug administration. The incidence of this event is quite low, with only two instances per million population per year. The sudden onset of necrosis in the epidermis is characterized by the shedding of skin to a greater or lesser extent [1,2].

Celecoxib is a non-steroidal anti-inflammatory drug (NSAID) that specifically inhibits cyclooxygenase-2 (COX-2) at the doses used in clinical practice (200-400 mg/day). COX-2 is the inducible isoform of cyclooxygenase, upregulated in response to pro-inflammatory stimuli, and is widely recognized as the principal source of the prostanoid mediators driving pain, inflammation, and fever. By selectively targeting this pathway, celecoxib delivers anti-inflammatory and analgesic effects while sparing the physiological functions mediated by other prostaglandin synthesis pathways.

It is commonly prescribed for alleviating arthralgia because of its superior gastrointestinal tolerability. Moreover, it may also cause cutaneous reactions, most of which are harmless. The medical literature has documented fewer than 10 instances of acute generalized exanthematous pustulosis (AGEP) [3-5]. TEN secondary to celecoxib is poorly documented.

In this report, we describe a case of Lyell syndrome (TEN) that occurred following the use of celecoxib. This case is reported because of the rarity of this severe reaction, the extensive use of celecoxib, and the need to raise clinicians' awareness of potentially life-threatening cutaneous toxicity, even with drugs generally considered safe.

## Case presentation

We present the case of a 69-year-old man who had been diagnosed with insulin-dependent diabetes and hypertension 15 years earlier. He was receiving insulin for diabetes management and the combination medication Exforge (amlodipine/valsartan) for hypertension control. The patient was admitted with a scarlet fever-like rash accompanied by skin detachment.

Investigations revealed that he had been taking celecoxib for six days, following a medical prescription, to treat arthralgia. The patient was subsequently admitted to the Dermatology Department, where he was hospitalized for seven days.

On admission, the patient was conscious, hemodynamically and respiratorily stable, with a BP of 130/70 mmHg, morbilliform erythroderma, and skin detachment involving <10% of the body surface area. Laboratory investigations showed the following: WBC, 9,500/mm³; urea, 1.44 g/L; creatinine, 21.1 mg/L; K+, 4.5 mmol/L; Na+, 128 mmol/L; sterile cytobacteriological examination of urine (CBEU); and no hepatic cytolysis. Therapeutic measures included celecoxib discontinuation, daily wound dressings, antibiotic therapy with amoxicillin/clavulanic acid, and thromboprophylaxis.

The following changes occurred seven days later: clinical worsening, disturbance of consciousness (Glasgow Coma Score (GCS) not specified), and biological deterioration, including impaired renal function (creatinine, 76 mg/L; urea, 2.48 g/L) and abnormal liver function tests (cytolysis and cholestasis: aspartate transaminase (ASAT), 2.5×N; alanine transaminase (ALAT), 2×N; gamma-glutamyl transferase (GGT), 8.8×N).

Given the clinical and biological deterioration, he was transferred to our Intensive Care Unit (ICU) for management.

**Figure 1:**
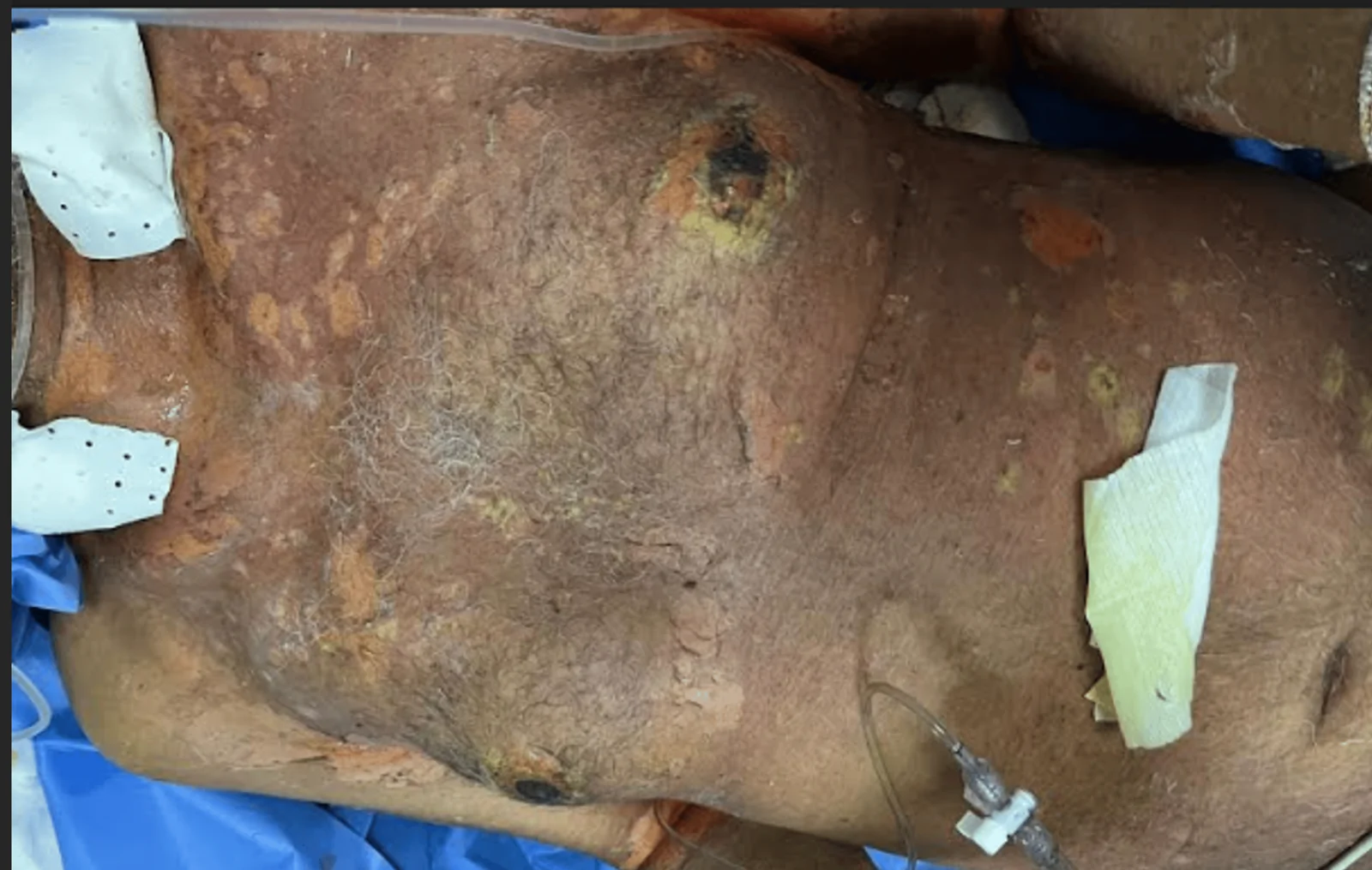
Lesions on the frontal part of the trunk

**Figure 2:**
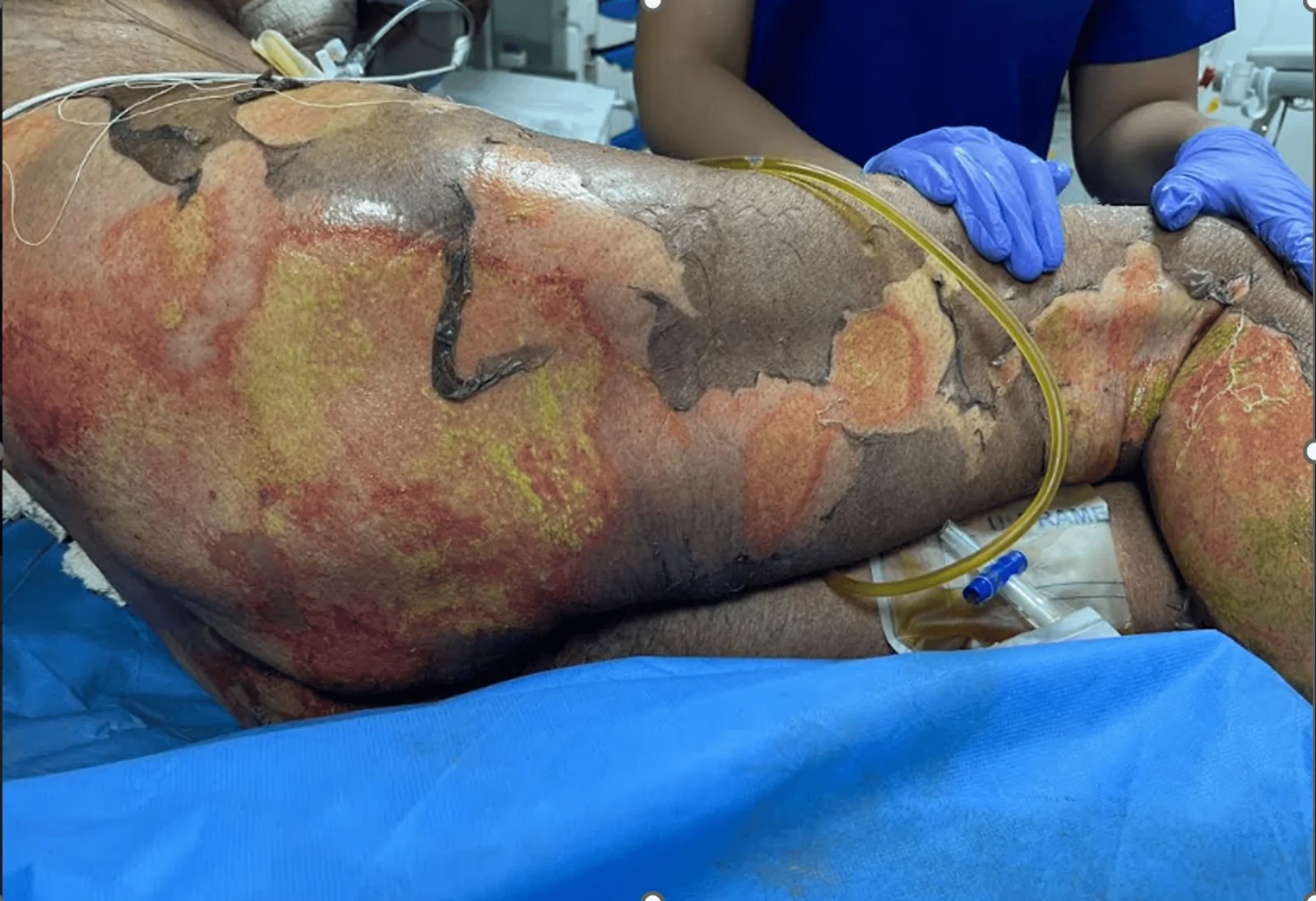
Characteristic moist denuded dermis with skin detachment from the gluteal region and upper thigh

**Figure 3:**
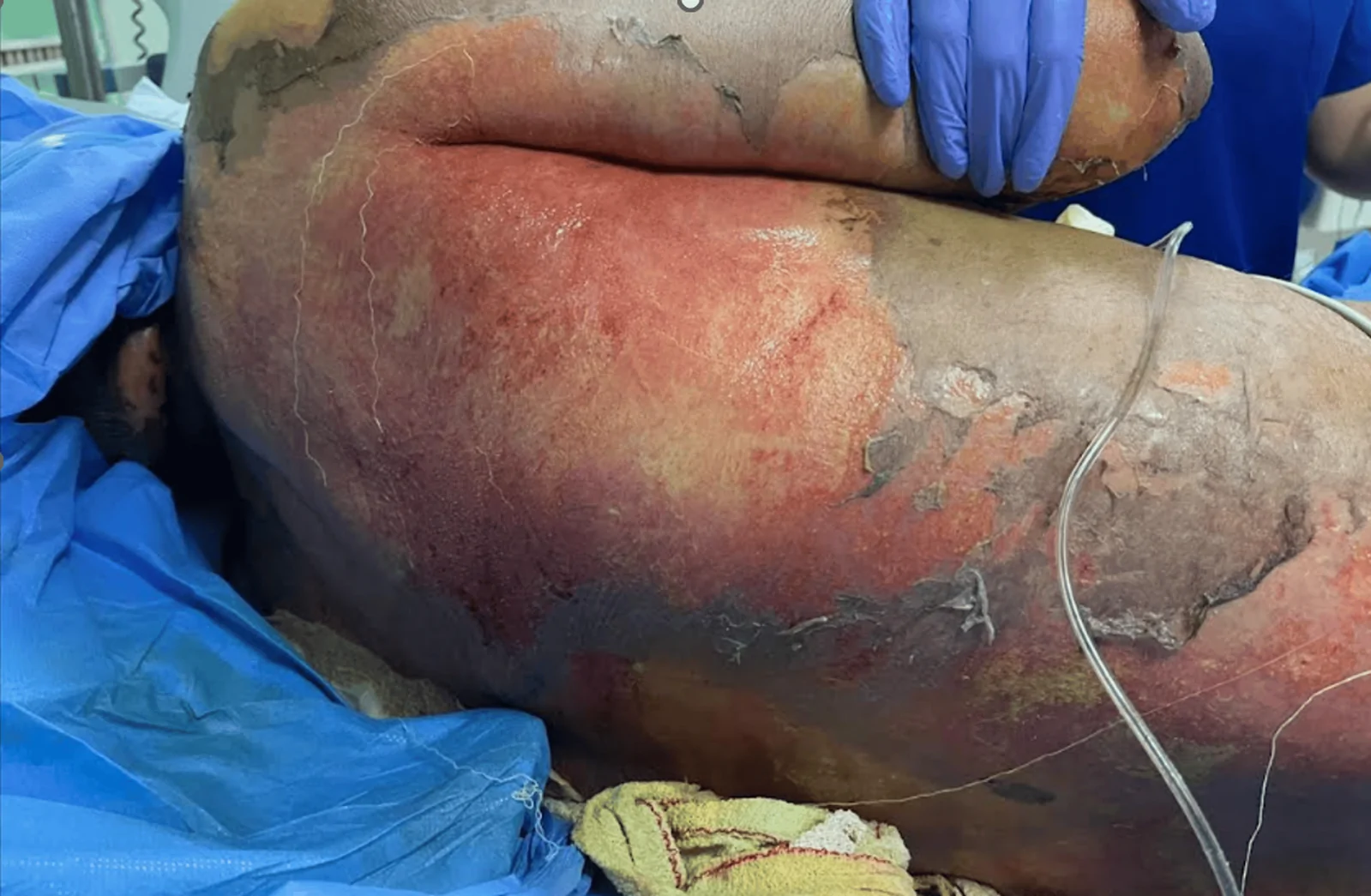
Characteristic moist denuded dermis of the back

Clinical examination on admission to our unit revealed an unconscious patient with a GCS of 11-12/15, BP of 80/40 mmHg, heart rate of 125 bpm, and SpO₂ of 91% on room air. A scarlatiniform exanthema covered 80% of the integument, with skin detachment involving 40% of the body surface area and a characteristic moist, denuded dermis (Figures 1-3). Oral enanthema and bilateral conjunctivitis with yellowish secretions were also present. The patient had an ALDEN score of 4 and a SCORTEN of 5.

Biological tests revealed the following abnormalities (Table 1): albumin, 17 g/L; CRP, 294.71 mg/L; procalcitonin, 21.27 ng/L; lactate dehydrogenase (LDH), 302 IU/L; and lymphocytes, 670/µL. 

**Table 1:** Biological assessment CRP: C-reactive Protein; LDH: Lactate Dehydrogenase; WBC: White Blood Cell; ASAT: Aspartate Amino-Transferase; ALAT: Alanine Amino-Transferase

Variable	Patient Results	Normal Value
Albumin	17 g/L	35 - 50 g/L
CRP	294.71 mg/L	0 - 5 mg/L
Urea	2.51 g/L	0.15 - 0.45 g/L
Creatinine	82.96 mg/L	7.2 - 12.5 mg/L
LDH	302 IU/L	125 - 243 IU/L
Procalcitonin	21.27 ng/L	< 0.1 ng/L
Hemoglobin	9.7 g/dL	12 - 18 g/dL
WBC	7340/µL	4000 - 10000/µL
Platelets	193000/µL	150000 - 400000/µL
Lymphocytes	670/µL	1000 - 4000/µL
ASAT	74 IU/L	5 - 34 IU/L
ALAT	58 IU/L	0 - 55 IU/L

Arterial blood gas (ABG) analysis showed the following (Table 2): pH, 7.39; PaO₂, 69 mmHg; PaCO₂, 30.5 mmHg; SaO₂, 94%; HCO₃⁻, 13.9 mmol/L; lactate, 3 mmol/L.

**Table 2:** Arterial blood gas pH: Potential of Hydrogen; PO_2_: Partial Pressure of Oxygen; PCO_2_: Partial Pressure of Carbon Dioxide; SaO_2_: Oxygen Saturation; HCO_3_^-^: Bicarbonate; AA: Ambient Air

Variable	Patient Results	Normal Values
pH	7.39	7.35 - 7.45
PaO₂	69 mmHg	75 - 100 mmHg
PCO_2_	30.5 mmHg	35 - 45 mmHg
SaO₂	94%	>95%
O_2 _(L/min)	AA	AA
HCO₃⁻	13.9 mmol/L	22 - 26 mmol/L
Lactate	3 mmol/L	< 2 mmol/L

**Figure 4:**
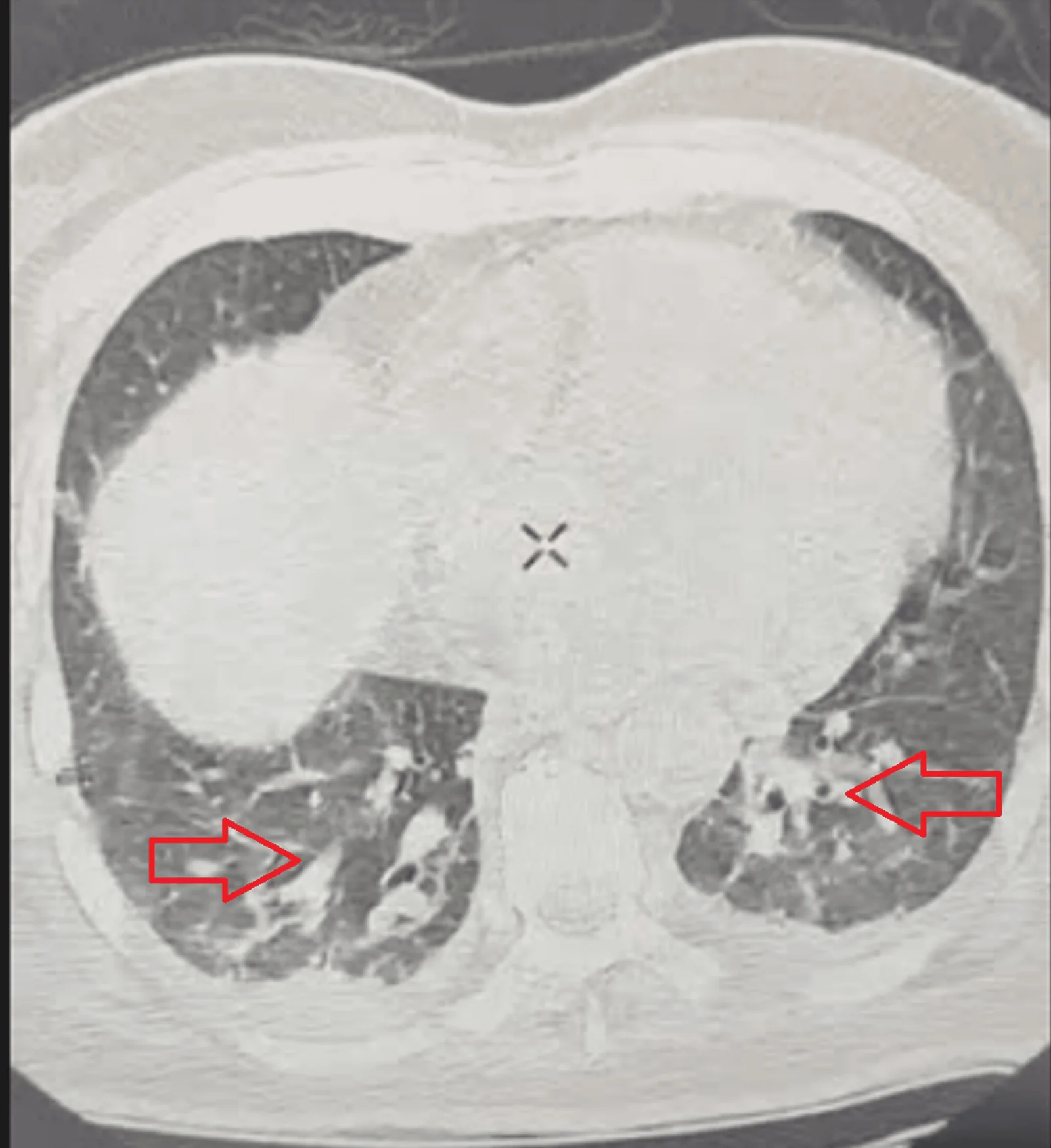
Cross-sectional image through the lung bases showing minimal bilateral basal pneumonia (red arrows)

The skin swab was sterile, and no biopsy was performed. The patient underwent a non-contrast CT scan of the thorax, which revealed bilateral infectious pneumonitis (Figure 4).

He was treated with rehydration according to Brooke's regimen, vasoactive drugs, antibiotics, albumin, oxygen therapy via nasal cannulas, daily wound dressing changes, a protein-rich diet, plasma exchange, and eye care. The outcome was unfavorable, and the patient died 48 hours after admission to the ICU.

## Discussion

Epidermal necrolysis includes Stevens-Johnson syndrome (SJS, <10% of the skin affected), Lyell syndrome (also known as TEN, with ≥30% of the skin affected), and overlap syndrome (10%-29% of the skin affected) [6,7].

The incidence of TEN is estimated at between two and seven cases per million population per year in the general population [8,9]. The absolute risk of SJS/TEN in new users of COX-2 inhibitors is 1.9 cases per 100,000 at first use [10].

According to the literature, TEN tends to occur more frequently in women, with a male-to-female ratio of 0.46 and a predominance in the sixth decade of life [11,12], whereas our patient was a 69-year-old man.

TEN is a delayed hypersensitivity reaction involving drug-specific CD4+ and, especially, CD8+ T lymphocytes after a 7-10-day sensitization phase. Upon re-exposure, these lymphocytes migrate to the skin and trigger keratinocyte death through perforin, granzyme B, granulysin, FasL, TRAIL, and TNF. Granulysin appears to play a central role, with its level in blister fluid strongly correlated with clinical severity. Drug-specific CD8+ T cells, sometimes showing oligoclonal expansion, have been identified in patients with TEN caused by carbamazepine or allopurinol. The first symptoms appeared six days after treatment was initiated. This is consistent with the typical time to onset of the clinical manifestations of TEN (4-28 days) after initiating a medication [1,6].

Clinically, skin involvement is sometimes the first manifestation of the disease, taking the form of ill-defined erythematous macules with purpuric centers or diffuse erythema, which may be tender to the touch. Lesions are often symmetrical, sparing the scalp and the palms and soles of the feet. They often begin on the face and then spread to the chest. There is often a positive Nikolsky sign, followed by the appearance of vesicles and bullae, which subsequently rupture. Mucosal involvement, commonly affecting the oral, ocular, and urogenital areas, is present in approximately 90% of patients. Ocular involvement often manifests as conjunctivitis with purulent discharge, corneal ulceration, anterior uveitis, panophthalmitis, and trichiasis [13-15]. Our patient was admitted with widespread, red, flat patches with skin detachment. Initially, the lesions were mainly on the chest and later spread to the thighs and calves, as shown in Figures 1-3. We also observed conjunctivitis.

The diagnosis is primarily clinical, and the ALDEN imputability algorithm can aid in diagnosis. There are no specific biological markers. Additional tests are conducted to exclude other potential diagnoses or identify accompanying organ dysfunction [6,8].

We performed the minimum emergency laboratory work-up in accordance with the recommendations of the 2023 PNDS (France) for adult epidermal necrolysis. Certain non-specific laboratory abnormalities are also markers of severity, including lymphopenia, hypophosphatemia, hypoalbuminemia, and elevated LDH. This patient had hypoalbuminemia, lymphopenia, and elevated LDH, all of which are markers of poor prognosis.

It is recommended that these patients be admitted to a burns center, even in cases of initially mild disease, as the condition can progress very rapidly [1,16].

TEN is a critical and urgent condition that requires multidisciplinary treatment, with close collaboration between specialists, including dermatologists, resuscitation experts, dietitians, psychiatrists, ophthalmologists, and ear, nose, and throat specialists. There are several aspects to the treatment, the first and most important of which is, of course, the withdrawal of all drugs suspected of having induced TEN. Other measures include dressing; rehydration according to the Brooke Nutrition formula; maintaining the room temperature between 28°C and 32°C; analgesia/anxiolytics; oxygen support or mechanical ventilation if breathing difficulties occur; and infection prevention, including protective isolation, antiseptic cleansing, impregnated catheters, and no routine antibiotic prophylaxis.

To date, supportive care remains equivalent or superior to the therapeutic options already considered, including systemic corticosteroid therapy, intravenous immunoglobulins, cyclosporine, anti-TNF agents, other immunosuppressants, and hematopoietic growth factors [1,6]. Ragragui Ouasmin et al. (2022) and Braud et al. (2023) found these therapies to be beneficial [17,18], whereas Bamichas et al. (2002) and Senda and Fushimi (2024) argued the opposite [19,20].

The overall prognosis of patients admitted to intensive care for multivisceral failure is very poor (mortality 57%) [8]. SCORTEN is used to assess vital risk in the acute phase, and an increase during hospitalization is a poor prognostic factor [1,6]. He had a predicted mortality of 90% with a SCORTEN of 5. He died 48 hours after hospitalization. Although the clinical presentation was highly suggestive, a skin biopsy would have been valuable to help exclude differential diagnoses.

This case underscores the importance of close monitoring and prompt recognition of TEN. Early identification of warning signs, including rapid skin progression, mucosal involvement, or systemic manifestations, is essential. Patients should be instructed to discontinue the suspected drug immediately and seek urgent medical attention.

## Conclusions

TEN is primarily a reaction induced by drugs. The diagnosis is essentially clinical. In this instance, the ALDEN score was 4 (i.e., a level of responsibility: probable). The prognosis depends on the degree of skin involvement and the presence of associated organ failure. Discontinuation of the suspected or causative drug(s), prompt transfer of the patient to a specialist unit, and provision of supportive care are the fundamental components of the patient's management.
